# Exceptionally preserved stomach contents of a young tyrannosaurid reveal an ontogenetic dietary shift in an iconic extinct predator

**DOI:** 10.1126/sciadv.adi0505

**Published:** 2023-12-08

**Authors:** François Therrien, Darla K. Zelenitsky, Kohei Tanaka, Jared T. Voris, Gregory M. Erickson, Philip J. Currie, Christopher L. DeBuhr, Yoshitsugu Kobayashi

**Affiliations:** ^1^ Royal Tyrrell Museum of Palaeontology, Drumheller, Alberta, Canada.; ^2^ Department of Earth, Energy, and Environment, University of Calgary, Calgary, Alberta, Canada.; ^3^ Graduate School of Life and Environmental Sciences, University of Tsukuba, Tsukuba, Ibaraki, Japan.; ^4^ Department of Biological Science, Florida State University, Tallahassee, FL, USA.; ^5^ Department of Biological Sciences, University of Alberta, Edmonton, Alberta, Canada.; ^6^ Hokkaido University Museum, Hokkaido University, Sapporo, Hokkaido, Japan.

## Abstract

Tyrannosaurids were large carnivorous dinosaurs that underwent major changes in skull robusticity and body proportions as they grew, suggesting that they occupied different ecological niches during their life span. Although adults commonly fed on dinosaurian megaherbivores, the diet of juvenile tyrannosaurids is largely unknown. Here, we describe a remarkable specimen of a juvenile *Gorgosaurus libratus* that preserves the articulated hindlimbs of two yearling caenagnathid dinosaurs inside its abdominal cavity. The prey were selectively dismembered and consumed in two separate feeding events. This predator-prey association provides direct evidence of an ontogenetic dietary shift in tyrannosaurids. Juvenile individuals may have hunted small and young dinosaurs until they reached a size when, to satisfy energy requirements, they transitioned to feeding on dinosaurian megaherbivores. Tyrannosaurids occupied both mesopredator and apex predator roles during their life span, a factor that may have been key to their evolutionary success.

## Teaser

A young tyrannosaur ingested two yearling dinosaurs, showing such predators hunted small prey but shifted to large prey as adults.

## INTRODUCTION

Tyrannosaurids are a clade of carnivorous dinosaurs that dominated the ecosystems of Asia and North America near the end of the Cretaceous period [~80 to 66 million years (Ma) ago] (*1*–*4*). Among the largest terrestrial predators to have ever existed, tyrannosaurids grew from meter-long hatchlings to multiton sizes (9- to 12-m long, 2000 to 6000 kg) over the course of their life span (*3*–*5*). Juveniles were gracile with narrow skulls, blade-like teeth, and long slender hind limbs, whereas adults were robust with massive skulls and large incrassate teeth and were capable of generating bone-crushing bites (*3*, *4*, *6*–*11*). These marked morphological changes suggest that tyrannosaurids underwent a major ontogenetic dietary shift, in which immature/juvenile and mature/adult individuals occupied different ecological niches (*7*, *10*, *12*, *13*). Fossil evidence reveals that dinosaurian megaherbivores (i.e., species with an adult mass > 1000 kg, including ceratopsids, giant ornithomimosaurs, hadrosaurids, and sauropods) were common prey items of large tyrannosaurids (*8*, *14*–*20*), a diet for which the necessary craniodental adaptations and bite forces only developed when individuals reached late juvenile or early subadult growth stages (*7*, *10*, *11*, *21*). Unfortunately, fossil evidence for diet in young tyrannosaurids is largely unknown, thus limiting our understanding of ontogenetic dietary shifts in these iconic predators.

**Fig. 1.**
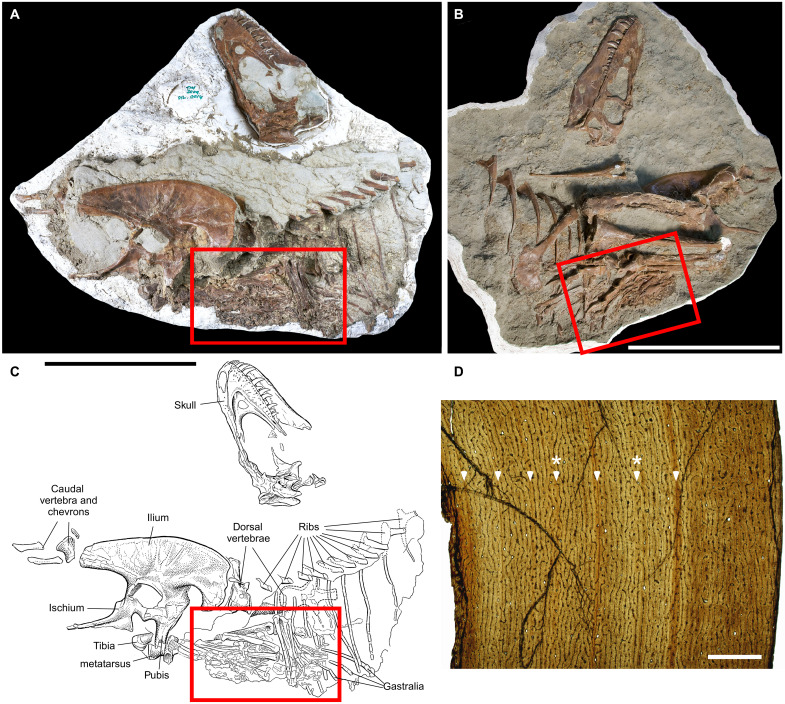
Juvenile *Gorgosaurus* TMP 2009.12.14 preserving stomach contents. Photographs of specimen in (**A**) right lateral view and (**B**) left anterolateral view. (**C**) Interpretive illustration of specimen in right lateral view. Skeleton consists of a nearly complete skull, the left side of the body and limbs, and a nearly complete pelvis. Red rectangle delineates location of stomach contents. (**D**) Histological photomicrograph of tibia showing the presence of five lines of arrested growths and two annuli (marked by asterisks), indicating that the individual was between 5 and 7 years old. Scale bars, 50 cm (A) to (C) and 1 mm (D).

Providing direct fossil evidence of diet and feeding behavior in young tyrannosaurids, here, we report on an articulated skeleton of a juvenile *Gorgosaurus libratus* from the Upper Cretaceous Dinosaur Park Formation (~75.3 Ma) of Alberta, Canada (see Supplementary Text), that preserves the remains of two small caenagnathid theropods (Oviraptorosauria) in its abdominal cavity (Fig. 1, A to C). This specimen [Royal Tyrrell Museum of Palaeontology (TMP) 2009.12.14] represents, to our knowledge, the first instance of in situ stomach contents (i.e., preserved in proper anatomical position) for a tyrannosaur and provides direct fossil evidence of diet and feeding behavior in a young tyrannosaurid.

## RESULTS

With an estimated body mass of 335 kg based on its femur length, the *Gorgosaurus* individual would have been less than 13% of the body mass of an adult conspecific (see Supplementary Text). Bone histology, specifically bone fabric and the presence of growth marks, reveals that the animal was a juvenile between 5 and 7 years of age at the time of its death (Fig. 1D; see Supplementary Text).

**Fig. 2.**
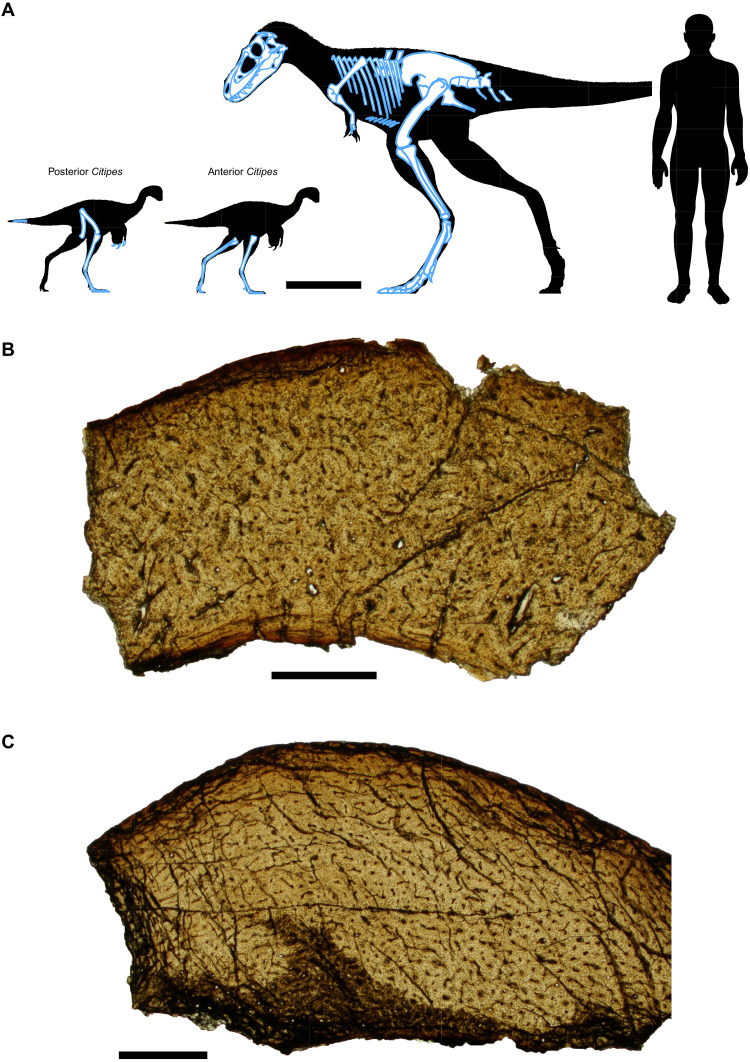
Juvenile *Citipes* remains preserved as stomach contents. (**A**) Diagram illustrating relative body sizes of predator and prey and skeletal elements preserved in TMP 2009.12.14. Scale bar, 50 cm. Histological photomicrographs of (**B**) posterior *Citipes* individual (metatarsal II) and (**C**) anterior *Citipes* individual (tibia), showing highly vascularized woven bone with reticular and longitudinally oriented vascular canals and lacking growth lines, indicative of young individuals that are less than 1 year old. Scale bars, 500 μm.

**Fig. 3.**
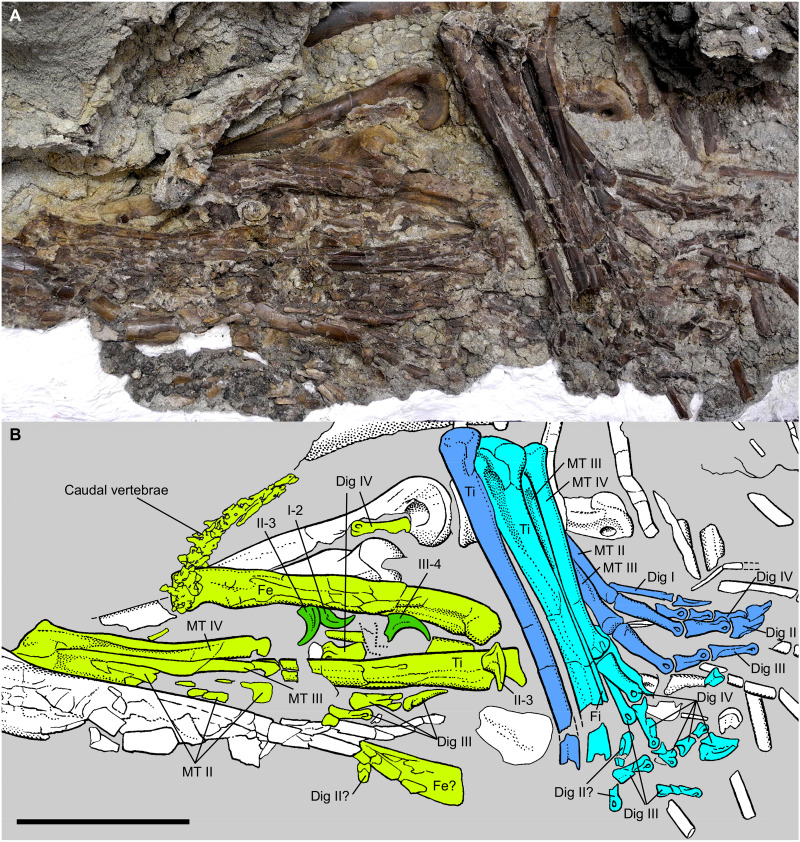
Stomach contents of juvenile *Gorgosaurus*. Photograph (**A**) and interpretive illustration (**B**) of stomach contents. Some of the caenagnathid bones are truncated and weathered due to modern erosion along the ventral abdominal region. Light blue and dark blue elements are the left and right hindlimbs, respectively, of the anterior *Citipes* individual. Light green elements are the hindlimbs and caudal vertebrae of the more posterior *Citipes* individual. Dark green elements represent the manual unguals of the posterior *Citipes* individual. White elements represent the juvenile *Gorgosaurus*. Fe, femur; Ti, tibia; Fi, fibula; MT, metatarsal; dig, pedal digit. Scale bar, 10 cm. See Supplementary Text for detailed identification.

Skeletal remains preserved in the abdominal cavity of the juvenile *Gorgosaurus* consist exclusively of articulated and associated postcranial elements, primarily from the hindlimbs, of two separate individuals of the small caenagnathid theropod *Citipes elegans* (Fig. 2A; see Supplementary Text for diagnostic characters). Bone histology, including bone fabric and the absence of growth marks, reveals that both *Citipes* individuals were within their first year of life (Fig. 2, B and C; see Supplementary Text). The *Citipes* remains are restricted to a small area (44-cm long by 23-cm deep) in the posterior portion of the abdominal cavity of the juvenile tyrannosaurid, extending between the 18th and 23rd dorsal ribs. These remains are immediately underlain by the ventral part of the left rib cage and a series of left gastralia (Fig. 3). The hindlimbs of both *Citipes* individuals are fully flexed, with the long bones of the legs and feet closely appressed. In addition to the location of the hindlimbs within the *Gorgosaurus* body cavity, the caenagnathid remains meet established criteria to be interpreted as stomach contents (table S1; see Supplementary Text). The extreme flexion of the hindlimbs suggests they were contained and compressed within the muscular stomach.

**Fig. 4.**
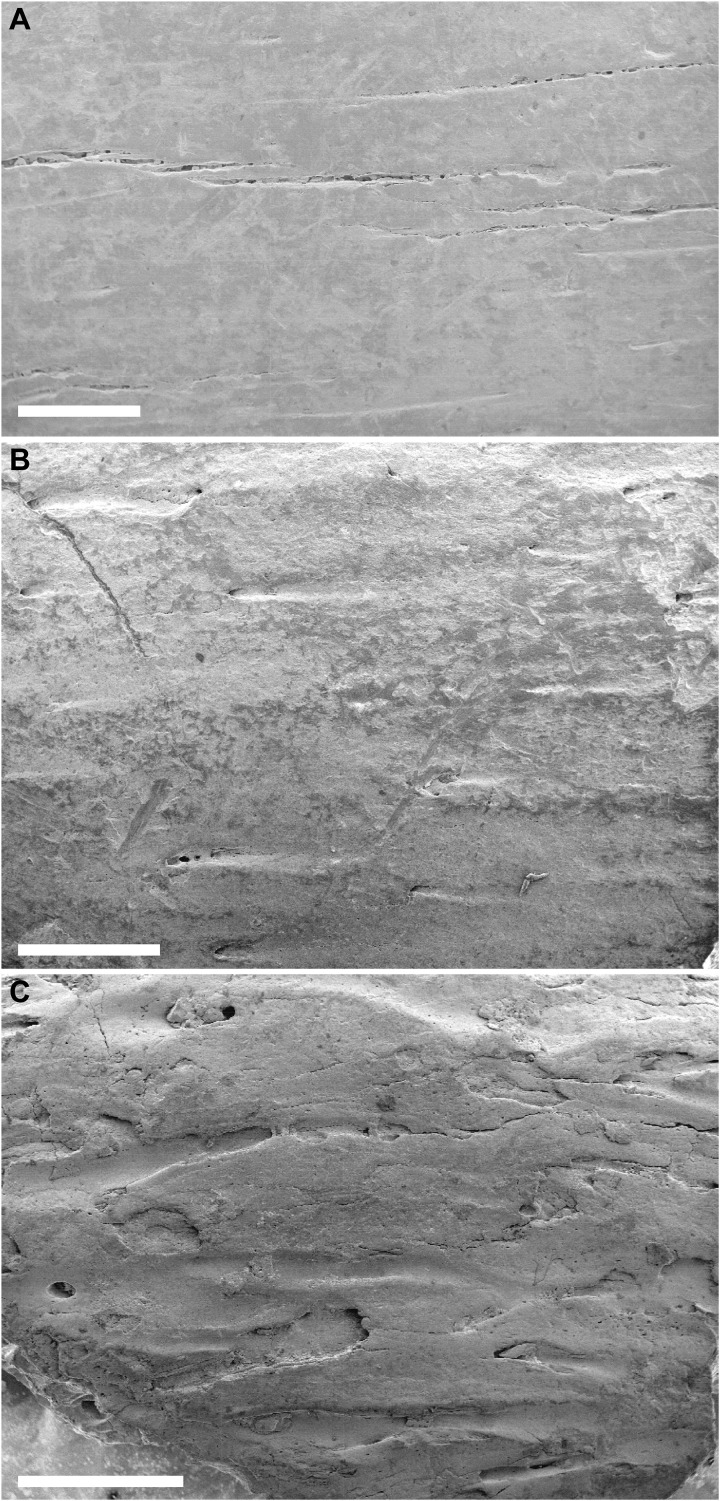
Scanning electron photomicrographs of bone surface texture. (**A**) *Gorgosaurus* specimen (tibia). (**B**) Anterior *Citipes* individual (phalanx III-2). (**C**) Posterior *Citipes* individual (phalanx IV-1). Whereas bone surface is smooth in the tyrannosaurid, it is tarnished in the anterior *Citipes* individual and visibly etched in the posterior *Citipes* individual as a result of exposure to low-pH gastric acids. The more extensive bone surface damage in the posterior *Citipes* individual reflects its longer residence time in the stomach than the anterior *Citipes* individual. Scale bars, 500 μm.

The location and orientation of the remains of the two *Citipes* individuals in the abdominal cavity differ, with the elements of one individual situated anterior and oriented perpendicular to those of the second individual (Fig. 3 and fig. S1). The anterior *Citipes* individual consists of both lower hindlimbs. The long bones are oriented almost dorsoventrally in the *Gorgosaurus* body cavity and are largely articulated, except along the erosional edge where the phalanges appear to have been disturbed by weathering. Both legs are flexed at the ankle and preserved side by side, with the right foot folded and partially concealed under the left leg. In the right foot, metatarsal and phalangeal elements are fully articulated, although digit IV is folded under digits II and III and its phalanges are exposed between those of digits I and II. In the left foot, metatarsal I and II and phalanges of their respective digits are not visible; metatarsals III and IV are visible in lateral view, with phalanges of digit III slightly scattered and those of digit IV preserved in articulation. The posterior *Citipes* individual consists of an articulated left leg and metatarsus folded at both the knee and ankle. The long bones are oriented anteroposteriorly in the abdominal cavity, with the femur lying above and roughly parallel to the tibia and metatarsus. Several isolated pedal phalanges, three isolated manual claws, and a short series of articulated caudal vertebrae (fig. S3; see Supplementary Text) are scattered among the limb bones of this individual. A highly weathered elongate element, likely the right femur of this individual, is situated along the erosional edge of the specimen. Consistent with stomach acid etching, the surface of the caenagnathid bones is altered compared to the smooth bone surface of the juvenile *Gorgosaurus*: The bone surface of the anterior *Citipes* individual appears tarnished, whereas that of the posterior individual is more extensively etched and pitted (Fig. 4).

**Fig. 5.**
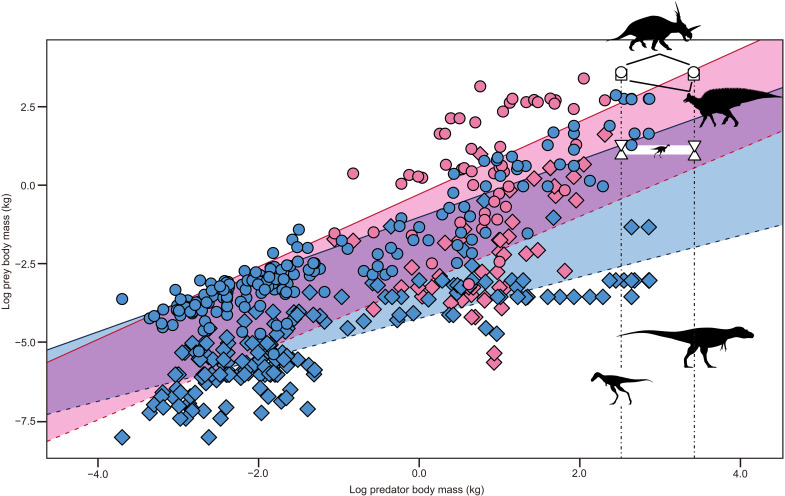
Phylogenetically corrected regressions of maximum and minimum prey mass against predator mass. Regression lines for extant terrestrial mammals are red [coefficient of determination (*R*^2^) = 0.24 and 0.25 for minimum and maximum prey regressions, respectively] and blue for extant reptiles (*R*^2^ = 0.31 and 0.66 for minimum and maximum prey regressions, respectively). Shaded areas represent prey size range of mammalian (red) and reptilian (blue) predators. Vertical dashed lines indicate body mass of juvenile (left) and adult (right) *Gorgosaurus*. Red circles, maximum prey mass of mammalian predators; blue circles, maximum prey mass of reptilian predators; red diamonds, minimum prey mass of mammalian predators; blue diamonds, minimum prey mass of reptilian predators; solid lines, maximum prey mass regressions for mammalian (red) and reptilian (blue) predators; long dashed lines, minimum prey mass regressions for mammalian (red) and reptilian (blue) predators; white triangles and inverted triangles, yearling and adult *Citipes* mass, respectively; white circles, adult ceratopsid mass; white squares, adult hadrosaurid mass. Silhouettes derived from Phylopic and other sources (see Acknowledgments).

Body size relationships between extant predators and their prey provide insight into the diet of *Gorgosaurus* and tyrannosaurids in general. In extant mammalian and reptilian predators, a statistically significant (*P* < 0.01) positive correlation between predator and prey mass indicates that both minimum and maximum prey sizes increase with predator size (Fig. 5; see Supplementary Text). Inclusion of *Gorgosaurus* in the dataset reveals that juvenile and adult *Citipes* fall confidently within the expected prey size range for the juvenile *Gorgosaurus*, whereas sympatric dinosaurian megaherbivores (e.g., ceratopsids and hadrosaurids) plot above that range, beyond the maximum prey size for any extant predator of juvenile *Gorgosaurus* size. In contrast, dinosaurian megaherbivores plot at the upper prey size limit for adult *Gorgosaurus* based on the mammalian predator regression and above the maximum prey size of extant crocodylians, whereas *Citipes* falls much lower (in the lower prey size range for extant predatory mammals and upper range for living predatory reptiles).

## DISCUSSION

### Feeding strategy of a young tyrannosaurid

The stomach contents of TMP 2009.12.14 provide insights into the distinctive feeding strategy of a juvenile tyrannosaurid. The presence of mainly articulated hindlimb elements from two different caenagnathid individuals indicates that the predator did not ingest the entire carcasses despite the small body size of its prey but rather selectively dismembered each prey item to ingest the well-muscled hindlimbs (Fig. 2A). This feeding habit is consistent with that of extant carnivorans and crocodylians, in which the hindquarters and viscera are usually consumed first (*22*–*24*). Prey dismemberment by the young *Gorgosaurus* might indicate that the size of its pharyngeal opening limited the size of elements that it could ingest, in contrast to large individuals of *Varanus komodoensis* (Komodo dragon) and crocodylians that are capable of ingesting sizeable prey in their entirety (*25*, *26*).

The relative location of the two *Citipes* individuals in the abdominal cavity of the tyrannosaurid suggests that the posterior individual was consumed first. The extent of acid etching and bone articulation indicate that neither individual was extensively digested at the time of the young tyrannosaurid’s death. The slightly greater extent of acid damage and disarticulation (i.e., phalanges completely disarticulated) of the posterior individual indicate that the two *Citipes* individuals were ingested in separate feeding events in close succession, with the posterior individual residing in the stomach for a slightly longer time interval (perhaps hours to days). If gastric conditions were similar to the low-pH conditions of extant crocodylians (*27*), then the nature and extent of acid etching on the bones of the *Citipes* individuals suggest that they resided in the stomach of the tyrannosaurid for a relatively short period (less than 1 week). The acid etching is much less extensive than that of dinosaur bones inferred to have spent a prolonged period of time (up to 13 days) in the stomach of non-avian theropods (*20*, *28*). Nevertheless, the presence of acid etching indicates that, like their adult counterparts (*20*, *29*, *30*), young tyrannosaurids digested the bones of their prey rather than regurgitating them, a behavior that likely evolved only among later-diverging paravian theropods (*31*).

The unique preservation of TMP 2009.12.14 reveals that this juvenile *Gorgosaurus* preyed on small, young individuals of a cursorial, herbivorous/omnivorous theropod species (*32*). With an estimated body mass of 9 to 12 kg, the ingested *Citipes* individuals were only ~45 to 60% of the body mass of an adult conspecific (see Supplementary Text). Bone histology confirms that both *Citipes* individuals were within their first year of life at the time of their death (Fig. 2, B and C). With two animals of the same species, age, and size ingested in separate events, the stomach contents suggest that the juvenile *Gorgosaurus* may have preferentially preyed upon young-of-the-year caenagnathids rather than as a case of circumstantial consumption. Preferential predation on young individuals or individuals of a particular species is a feeding strategy frequently used by extant predators (*33*, *34*).

### Ontogenetic dietary shift in *Gorgosaurus* and tyrannosaurids

The predator-prey body size relationships revealed in this study (Fig. 5) indicate that *Gorgosaurus* (and probably other tyrannosaurids) underwent a major ecological and dietary shift over the course of their life span. Large *Gorgosaurus* individuals are known to have consumed dinosaurian megaherbivores (*17*), but these would have been much too large for juvenile individuals (Fig. 5). Juvenile *Gorgosaurus* likely preyed on small dinosaurs, like *Citipes* and other similar-sized species [e.g., caenagnathids (*35*), orodromine ornithopods (*35*, *36*), and pachycephalosaurids (*35*)], which would rarely have been selected by adult individuals due to their small size/low energy value (*37*). Such prey could also have allowed these juvenile predators to avoid dangerous antagonistic interactions with megaherbivores (e.g., ceratopsids and hadrosaurids), many of which lived in multigenerational herds for protection (*38*). Although it has been suggested that tyrannosaurids may have hunted large prey in multigenerational packs (*39*), the current discovery, albeit a single specimen, reveals that this juvenile *Gorgosaurus* had hunted small prey, likely too small to be shared with conspecifics.

Previously inferred on the basis of anatomical features (*7*, *10*, *12*) and ecological modeling (*13*, *37*, *40*), an ontogenetic dietary shift is now supported in tyrannosaurids based on direct dietary evidence from TMP 2009.12.14. Ontogenetic dietary shifts are widespread in the animal kingdom, being common in invertebrates, fishes, amphibians, and reptiles but rarer among animals that undergo an extended period of parental care or live in groups where food is acquired by adults (e.g., mammals and birds) [for a review, see (*41*–*43*)]. Although dietary shifts can be associated with metamorphosis or changes in habitat use during ontogeny, changes in body size (and its associated metabolic consequences) are arguably the most important driver, particularly in animals that grow by several orders of magnitude during their life span (*42*, *43*). Ontogenetic dietary shifts are observed among large extant reptiles, such as Komodo dragons (*25*, *44*) and crocodylians (*26*, *45*, *46*), in which invertebrate and small vertebrate prey are the staples of juvenile individuals and large terrestrial vertebrates (mainly ungulate mammals) become the primary food source as individuals grow. These dietary shifts are often associated with changes in craniodental morphology (i.e., increase in skull and tooth robusticity), bite force, and methods of prey capture and food processing (*25*, *45*–*50*). A major change in prey size through ontogeny reflects the fact that the energy balance associated with hunting small animals (i.e., the energy requirements associated with capturing small, albeit abundant prey versus the amount of energy it provides the predator) becomes negative with increasing predator body size and thus requires the predator to undergo a dietary shift to larger prey to meet its energy requirements (*37*, *51*). Energetic models predict that young theropods would have transitioned from feeding primarily on prey smaller than themselves (e.g., insects, amphibians, lizards, mammals, and birds) to feeding on prey of their own body size (e.g., small dinosaurs) once they reached 16 to 32 kg (*37*). However, TMP 2009.12.14 reveals that juvenile *Gorgosaurus* still fed upon small dinosaur species (*Citipes* adult body mass ~20 kg; see Supplementary Text) even at a body mass of 335 kg. This suggests that small prey remained an important part of the diet of these juvenile predators long after they exceeded the predicted mass threshold (i.e., 16 to 32 kg) for feeding on prey as large as themselves. Craniodental adaptations and bite force estimates suggest that the dietary shift to feeding on dinosaurian megaherbivores in *Gorgosaurus* began gradually as individuals approached 600 kg [subadult stage, ~11 years old (*10*); see Supplementary Text], nearly twice the mass of TMP 2009.12.14. As such, the narrow, gracile skull and blade-like teeth of TMP 2009.12.14 and other juvenile tyrannosaurids (*7*, *10*, *11*) were ideally suited for capturing and dismembering small prey (like *Citipes*), whereas the broad, massive skulls and teeth of larger individuals were adapted for restraining large prey, biting through bone, and scraping and tearing flesh from carcasses (*8*–*10*, *17*, *52*–*54*).

### Ecological and evolutionary implications

The ontogenetic dietary shift recognized in *Gorgosaurus* provides insight into aspects of tyrannosaurid paleoecology and potential evolutionary drivers in these large predators. In modern ecosystems, ontogenetic dietary shifts provide a competitive advantage when prey for juvenile predators is in greater abundance than prey for adults as it can lessen intraspecific competition for resources (*42*); thus, such a dietary shift may have allowed juvenile and adult tyrannosaurids to coexist in the same ecosystem with limited conflict. Young dinosaurs, like yearling *Citipes*, could have represented an abundant and reliable food source for juvenile *Gorgosaurus* as oviraptorosaurs are known to have laid large egg clutches [>30 eggs per clutch (*55*)]. Very young tyrannosaurids likely competed with sympatric deinonychosaurs (i.e., generally ≤3-m-long, sickle-clawed dromaeosaurids and troodontids) for small prey in their ecosystems, although competition would have decreased as tyrannosaurid individuals aged and outgrew deinonychosaurs to become mid-sized predators. Species of mid-sized carnivorous dinosaurs (i.e., “mesopredators”) are rare or absent in latest Cretaceous terrestrial ecosystems of Asiamerica, where immature tyrannosaurids are considered to have filled these vacant ecological niches (*13*, *40*). As they grew beyond the juvenile stage, tyrannosaurids underwent a major dietary shift, from feeding on small prey to feeding on megaherbivores, likely in association with the development of robust craniodental anatomy in adults. Tyrannosaurid individuals thus transitioned from a mesopredator to an apex predator role, occupying both ecological niches over the course of their lifetime (*10*, *12*, *13*, *38*, *56*–*58*). A similar clade-wide ecological trend occurred in the evolutionary history of tyrannosauroids: These predators usually occupied the mesopredator niche between the Late Jurassic and early Late Cretaceous and evolved to become large apex predators in the later part of the Cretaceous after the extinction of large allosauroids, the long-reigning apex predators in Asiamerica (*59*–*61*). Through accelerated growth rates and extended growth duration (*62*), tyrannosauroid species were able to achieve large body size and develop robust craniodental anatomy, enabling them to evolve and take over the vacant apex predator niche. The ability of tyrannosauroids, including tyrannosaurids, to assimilate the apex predator ecological niche while retaining the ancestral mesopredator niche (as juveniles) was likely key to their evolutionary success as some of the largest carnivorous theropods to have existed.

## MATERIALS AND METHODS

### Institutional abbreviations

CMN, Canadian Museum of Nature, Ottawa, Ontario, Canada; TMP, Royal Tyrrell Museum of Palaeontology, Drumheller, Alberta, Canada.

### Histology

Histological thin sections of bone fragments were made by Calgary Rock and Materials Services, Calgary, Alberta, Canada, and examined under a Leica DM2500P polarizing microscope. Detailed descriptions are provided in Supplementary Text.

### Bone surface texture

Bone surface textures were documented with a FEI Quanta FEG 250 field emission scanning electron microscope operating under high vacuum conditions with an accelerating potential of 1 kV. Detailed descriptions are provided in Supplementary Text.

### Digital rendering of caudal vertebrae

The matrix block containing the *Citipes* caudal vertebrae was subjected to computed tomography (CT) on a Toshiba Aquilion medical CT scanner at the Drumheller Health Centre in Drumheller, Alberta, Canada. CT scanning was conducted at a voltage of 120 kV, an x-ray tube current of 300 mA, and with contiguous slices of a thickness of 0.5 mm. Dicom files were imported into the software Amira v.2019.1, and bones were digitally isolated from the matrix using a threshold mask and digitally rendered as an isosurface digital model. Dicom files are available on the online database MorphoSource.

### Predator-prey mass regressions

To assess the likelihood of *Gorgosaurus* preying on *Citipes*, the influence of body mass on predator-prey mass relationships was investigated in extant terrestrial mammalian and reptilian predators. Maximum and minimum prey mass was compiled for a range of terrestrial reptilian and mammalian predators based on the literature. Data for terrestrial mammals were gathered from Tucker and Rogers (*63*) and those of non-varanid lizards were from Costa *et al.* (*64*) (see data S1). Because the latter reported prey size in terms of volume, we transformed body volume into body mass by assuming body density close to that of water (1 ml = 1 g). Information regarding maximum and minimum prey mass and predator mass for extant varanid and crocodylian species was gathered from various sources (*25*, *44*, *65*–*83*) and is reported in data S1. Although Drumheller and Wilberg (*69*) listed the small prey hunted by each crocodilian species, they did not report minimum prey mass; for this reason, we used mass values for equivalent prey species reported in the aforementioned varanid diet literature. It is worth mentioning that the smallest prey reported for large reptiles, particularly crocodylians and large varanids, are not usually ingested by mature predators but by juvenile individuals as these species undergo an ontogenetic dietary shift, feeding on small prey when young and shifting to large prey as they grow (*25*, *26*, *44*–*46*).

To take into consideration the phylogenetic relationships of predators, phylogenetically corrected least-squares (PGLS) regressions of maximum and minimum prey mass against predator mass were conducted for reptilian and mammalian predators. Phylogenetic trees of mammals and reptiles were constructed on the basis of the works by Nyakatura and Bininda-Emonds (*84*), Pyron *et al.* (*85*), and Drumheller and Wilberg (*69*) (figs. S4 and S5 and data S2 and S3). Branch length was calculated from divergence time, which was taken from Nyakatura and Bininda-Emonds (*84*), Drumheller and Wilberg (*69*), and TimeTree (timetree.org; data retrieved from 6 to 13 August 2020), following the procedure of Motani and Schmitz (*86*). Constructed trees are ultrametric. Phylogenetic models with maximum-likelihood estimations of lambda were analyzed with R v.4.04 using the package caper v.1.0.1 (*87*). For comparison with PGLS regression, ordinary non-phylogenetic least-squares regressions were performed using IBM SPSS v.25. Additional information is provided in Supplementary Text.

### Measurements

All skeletal elements were measured with digital calipers (see Supplementary Materials).

## Supplementary Materials

### This PDF file includes:

Supplementary Materials and Methods Supplementary Text Figs. S1 to S5 Tables S1 to S4 Legends for data S1 to S3 References
