# Supplementary material for: Exceptionally preserved stomach contents of a young tyrannosaurid reveal an ontogenetic dietary shift in an iconic extinct predator
Source: Sci Adv. 2023 Dec 8;9(49):eadi0505. doi: 10.1126/sciadv.adi0505 (PMC10846869; doi:10.1126/sciadv.adi0505)
Supplement: Supplementary file 1 — Supplementary Materials and Methods Supplementary Text Figs. S1 to S5 Tables S1 to S4 Legends for data S1 to S3 References [file sciadv.adi0505_sm.pdf]

Science Advances
AAAS

Supplementary Materials for

Exceptionally preserved stomach contents of a young tyrannosaurid reveal an ontogenetic dietary shift in an iconic extinct predator

François Therrien *et al.*

Corresponding author: François Therrien, francois.therrien@gov.ab.ca; Darla K. Zelenitsky, dkzeleni@ucalgary.ca

Sci. Adv. **9**, eadi0505 (2023)
DOI: 10.1126/sciadv.adi0505

The PDF file includes:

Supplementary Materials and Methods
Supplementary Text
Figs. S1 to S5
Tables S1 to S4
Legends for data S1 to S3
References

Other Supplementary Material for this manuscript includes the following:

Data S1 to S3

Materials and Methods

Measurements

All skeletal elements were measured with digital calipers.

Histology

Histological thin sections of bone fragments were made by Calgary Rock and Materials Services, Calgary, Alberta, Canada and examined under a Leica DM2500P polarizing microscope.

Bone surface texture

Bone surface textures were documented with a FEI Quanta FEG 250 field emission scanning electron microscope operating under high vacuum conditions with an accelerating potential of 1 kV.

Digital rendering of caudal vertebrae

The matrix block containing the *Citipes* caudal vertebrae was subjected to computed tomography (CT) on a Toshiba Aquilion medical CT scanner at the Drumheller Health Centre in Drumheller, Alberta, Canada. CT scanning was conducted at a voltage of 120 kV, an X-ray tube current of 300 mA, and with contiguous slices of a thickness of 0.5 mm. Dicom files were imported into the software Amira v.2019.1 and bones were digitally isolated from the matrix using a threshold mask and digitally rendered as an isosurface digital model.

Institutional abbreviations

CMN, Canadian Museum of Nature, Ottawa, Ontario, Canada; TMP, Royal Tyrrell Museum of Palaeontology, Drumheller, Alberta, Canada.

Supplementary Text

1. Specimen locality

Specimen TMP 2009.12.14 was discovered in the badlands of Dinosaur Provincial Park, Newell County, Alberta, Canada (Quarry Q257). Exact geographic location data are archived in the collections of the Royal Tyrrell Museum of Palaeontology, Drumheller, Alberta, Canada. The specimen was found at the base of a light gray, trough cross-stratified sandstone in the upper "muddy interval" of the Dinosaur Park Formation (*88*), approximately 7 m below the stratigraphically lowest coal bed of the Lethbridge Coal Zone. As such, the specimen is stratigraphically bracketed by the "Plateau Tuff" and "LCZ" bentonites that have been radiometrically-dated at 75.639 ± 0.025 Ma and 75.017 ± 0.020 Ma, respectively (*89*). Following the Bayesian model median age-stratigraphic model for the Dinosaur Park Formation derived by Ramenazi et al. (*89*) in their figure 4, the strata containing TMP 2009.12.14 are estimated to be ~75.3 million years old.

The host sandstone is a multi-meter thick, lithic arenite that contains common gray mudclasts (up to pebble size) at the base. Localized sideritized lenses and laminations occur in the bed throughout the area. Mudclasts and disarticulated hadrosaur postcranial bones are dispersed at the base of the sandstone but microvertebrate remains (e.g., champsosaur vertebrae, turtle scutes, fish scales, crocodile and dinosaur teeth, small dinosaur bones), fossils otherwise frequently found in sandstones of the Dinosaur Park Formation, are absent. Given the sedimentological evidence, the specimen is interpreted to have been buried at the bottom of a river channel amid lag deposits.

2. Specimen taphonomy

TMP 2009.12.14 was buried lying on its left side. Whereas the left side of the animal is fully articulated and complete, the right side of the animal, including limbs and ribs, and many

vertebrae (except for one displaced posterior dorsal vertebra), are missing. This suggests that the left side of the skeleton was entombed in sediment, while the right side of the animal would have remained exposed (or been re-exposed to some extent after burial) such that these elements were lost.

Pebble-sized mudclasts occur in the matrix both inside and outside of the ribcage of the juvenile tyrannosaur individual. However, in the posterior region of the abdomen where the localized concentration of caenagnathid bones occurs, mudclasts are notably absent from the sediment. This difference in matrix infill, combined with the lack of caenagnathid bone or microvertebrate remains elsewhere inside or outside the ribcage, support the interpretation that the caenagnathid bones were contained within the walls of the digestive tract when the tyrannosaur was buried. Furthermore, the Dinosaur Park Formation is strongly biased against the preservation of small dinosaurs, so articulated remains of small theropods are rare (*35*). Although isolated metatarsi and other isolated bones of caenagnathids are known from the Dinosaur Park Formation (see (*90*)), articulated legs of these animals are extremely rare (*91, 92*). The discovery of articulated legs from two *Citipes* individuals in TMP 2009.12.14 provides strong evidence that these remains were preserved as such due to protection by the alimentary canal within the body cavity.

In their study of *in situ* stomach contents of an ankylosaur, Brown et al. (*93*) established a list of 16 independent criteria to identify stomach contents. Although some of the criteria only apply to herbivorous animals, most are applicable regardless of diet. Evaluation of these criteria reveals that the caenagnathid remains present in TMP 2009.12.14 meet nearly all of the criteria to qualify as stomach contents (Table S1).

3. Taxonomic affinity and histologic/ontogenetic assessment of skeletal remains

3.1. Tyrannosaurid

Two tyrannosaurid taxa co-occur in the Dinosaur Park Formation, the albertosaurine *Gorgosaurus libratus* and the tyrannosaurine *Daspletosaurus torosus*. Both tyrannosaurid taxa have been studied extensively and can be differentiated based on a suite of anatomical features (*1, 3, 6, 11, 94, 95*). TMP 2009.12.14 can be unequivocally identified as a juvenile individual based on bone histology and small body size (~4 m estimated body length, which is less than half the length of an adult of either sympatric tyrannosaurid taxon). The skull of this specimen was recently described in detail and found to be unequivocally identifiable as *Gorgosaurus libratus* based on the presence of autapomorphies of the species and synapomorphies of the subfamily Albertosaurinae (*11*). Three autapomophies of *Gorgosaurus* (*1, 3, 11, 94*) are present in TMP 2009.12.14: 1) the base of the postorbital process of the jugal is shorter than the minimum suborbital depth of the jugal, 2) the lateral frontal processes of the nasal extend further posteriorly than the medial processes, and 3) the frontals are dorsally convex and slope away from the interfrontal suture. The specimen also exhibits the following albertosaurine synapomorphies (*1, 3, 11, 94, 95*): 1) the posterior ramus of the postorbital terminates anterior to the posterodorsal margin of the laterotemporal fenestra; 2) the ventral margin of the jugal ramus of the quadratojugal is inclined anterodorsally; and 3) the ischium exhibits a subtle anterior flexure. Furthermore, TMP 2009.12.14 can be distinguished from the sympatric tyrannosaurine *Daspletosaurus torosus* based on several additional features (*1, 3, 11, 94-96*): 1) supranarial processes of left and right premaxilla separated by long processes of the nasal ; 2) 14 maxillary alveoli; 3) long caudolateral process of the nasal; 4) postorbital contact with the squamosal that extends anterior to the anterior margin of the laterotemporal fenestra; 5) ventral ramus of postorbital is curved anteriorly and tapers strongly ventrally; and 6) a narrow prefrontal. As demonstrated by Voris et al. (*11*), all available evidence indicates the tyrannosaur skeleton is assignable to *Gorgosaurus libratus*.

Histological analysis of the tibia of the *Gorgosaurus* specimen (Figure 1D in main text) reveals the bone is characterized by the presence of woven bone, the absence of erosion on the endosteal surface (erosion related to growth), and a predominance of circumferential and reticular vascularization, with few longitudinal vascular canals, all typically found in juvenile individuals (*97*). Haversian remodeling is absent, also indicative of a young individual. Lacunae are narrower than in the *Citipes* individuals (see below). The tibial cross-section displays five well-developed lines of arrested growth and two annuli in thin section, indicating that the animal was between 5 and 7 years of age at the time of its death (Fig. 1D in main text). As the spacing between these growth markers increases towards the periphery, it indicates that: 1) the animal was still a juvenile; 2) its absolute growth rate was increasing from year to year; and 3) it had not reached somatic maturity at the time of its demise.

3.2 Stomach contents

Postcranial skeletal elements are restricted to a small region of the abdominal cavity of the juvenile *Gorgosaurus* (Figures 1 and 3 in main text, fig. S1). Although examination is hindered by crushing and the bones being encased in matrix, the taxonomic identity of the stomach contents can be determined based on a combination of characteristics. Both individuals can be unequivocally referred to the caenagnathid *Citipes elegans* based on the presence of the following characteristics (*98*): 1) anterior surface of metatarsal III has longitudinal sulcus (visible in anterior individual, not possible to determine in posterior individual; fig. S2A); 2) distal condyle of metatarsal III is deeper than wide (visible in anterior individual, not possible to determine in posterior individual); 3) presence of a large posterolateral ridge on metatarsal IV (visible in both anterior and posterior individuals); and 4) long and gracile tarsometatarsus with length-to-width ratio of 6.25, as opposed to 4.55 for *Chirostenotes* (posterior individual has ratio of 6.16, but this is due to widening of the metatarsus caused by crushing; the ratio would be higher had it not been crushed; impossible to readily determine the ratio in the anterior individual). Furthermore, other features are similar to those observed in *Chirostenotes* and clearly indicate the specimens are caenagnathids: 1) shape of digit 1, consisting of a long, tubular metatarsal I and elongate ungual I-1 (visible in anterior individual; fig. S2B,C); 2) shape of manual unguals with deep “pendant”, especially one with a pronounced flexor tubercle similar to that typically seen on ungual III-4 of caenagnathids (visible in posterior individual; fig. S3A); and 3) shape of pedal ungual, which is short, robust, gently curved and proximally swollen (visible in posterior individual; fig. S3B). For all of these reasons, the stomach contents in TMP 2009.14.12 are identified as belonging to the small caenagnathid *Citipes elegans*.

Associated with the posterior specimen, a short series of caudal vertebrae was too delicate to be mechanically prepared. Instead, a matrix block containing the elements was extracted and subjected to computed tomography (CT) on a Toshiba Aquilion medical CT scanner at the Drumheller Health Centre in Drumheller, Alberta, Canada. Following previously established protocols (*99*), scans were conducted at a voltage of 120 kV, an X-ray tube current of 300 mA, and with contiguous slices of a thickness of 0.5 mm. DICOM files were imported into the software Amira v.2019.1 and digitally rendered as an isosurface digital model. Although the bones are thin and fragmented, an articulated series of several caudal vertebrae can be recognized (fig. S3C), some being superficially similar to the posterior-most vertebrae of the oviraptorosaur *Heyuannia yanshini* illustrated by Persons *et al*. (*100*).

Histological thin sections of limb elements of both *Citipes* individuals (tibia fragment from the anterior individual and metatarsal II fragment from the posterior individual) were studied in order to determine the ontogenetic stage of each individual. The tibia of the anterior *Citipes* individual is characterized by the presence of woven bone and absence of lines of arrested growth, typical of juvenile individuals (*97*) (Figure 2C in main text). Between the endosteal

surface and the periphery in the tibia, vascularization changes from longitudinal, to longitudinal and reticular, to circumferential. Lacunae are large and bulbous near the center of the bone, indicative of a young individual, but decrease in size towards the periphery of the bone, suggesting reduction in growth rate and may indicate the animal approached 1 year of age at the time of death.

As in the anterior *Citipes* individual, the metatarsal of the posterior *Citipes* individual is characterized by the presence of woven bone and the absence of lines of arrested growth, again typical of juvenile individuals (Figure 2B in main text). The bone displays a mixture of longitudinal and reticular vascularization, and vascularization does not diminish substantially from the center of the bone towards periphery. Lacunae are bulbous, indicative of a juvenile individual (*97*). Endosteal bone is present, and no lines of arrested growth or lamellae are present in the endosteal bone. A sinuous surface breakage is observed on both the exterior and endosteal surface of the bone, which could be indicative of chemical dissolution due to acid etching. Like the previous *Citipes* individual, this individual is estimated to have approached 1 year of age at the time of death.

4. Acid etching of bone surface

Study of bone surface texture was conducted using a FEI Quanta FEG 250 field emission scanning electron microscope (SEM) operating under high vacuum conditions with an accelerating potential of 1 kV. The tibia of the juvenile *Gorgosaurus* exhibits a very smooth bone surface, both to the naked eye and under SEM (Figure 4A in main text). In contrast, the bone surfaces of the *Citipes* individuals exhibit different degrees of roughness, both to the naked eye and under SEM. Phalanx III-2 of the anterior individual exhibits a tarnished surface (Figure 4B in main text) whereas phalanx IV-1 of the posterior individual exhibits an etched and pitted surface (Figure 4C in main text). The extent of bone surface damage during digestion is dependent on several factors, including pH of gastric acids and residence time in the stomach (*30*). Although the amount of time the *Citipes* individuals had resided in the stomach of the *Gorgosaurus* cannot be accurately determined, it is likely that they spent no more than a few days as bones are known to fully dissolve within 4-14 days in the stomachs of crocodylians depending on ambient temperature (*101*). Furthermore, the acid etching is also much less extensive than that observed on bones inferred to have spent a prolonged period of time (up to 13 days) in the stomach of theropods (*20*, *28*). Given the differences in acid etching between the anterior and posterior *Citipes* individuals, it can be inferred that the latter was ingested some time (hours to days) prior to the former.

5. Body mass estimation

Although some of the most frequently used body mass estimation methods rely on femur circumference (*102*, *103*), those require complete and largely undistorted elements to compute. Given the fact that the femora of the specimens are either crushed (juvenile *Gorgosaurus*, posterior *Citipes* individual) or absent (anterior *Citipes* individual), use of their circumference was not possible. Instead, the method of Christiansen and Fariña (*104*), which relies on femoral length, was used to estimate body mass: body mass (in kg) = -6.288 + 3.22*LOG(femur length, in mm) (see Table S2). This method was shown to be consistent with body mass estimates obtained from scaling methods based on the circumference of stylopodial elements in extant terrestrial tetrapods (*105*). The length of the femur of the anterior *Citipes* individual was estimated based on the length ratio of metatarsal IV/femur measured in the posterior individual, which has a complete femur. For comparative purposes and consistency of methodology, the body masses of adult *Gorgosaurus* (CMN 2120), subadult *Gorgosaurus* (TMP 1991.36.500), and adult *Citipes* (TMP 1982.16.6) individuals were estimated using the method of Christiansen and Fariña (*104*) as well (Table S2). A volumetric-density model has previously estimated the body mass of the adult

Gorgosaurus CMN 2120 at 2427 kg (*106*), which is only 7.6% lighter than the body mass estimate we obtained and provides additional support for the method of Christiansen and Fariña (*104*).

Based on the results in Table S2, it is possible to compare the body mass of the various theropod individuals in TMP 2009.12.14. The juvenile *Citipes* individuals are ~3% of the body mass of the juvenile *Gorgosaurus* and ~46.5-58.1% of the body mass (~79-84.5% of metatarsal length) of an adult *Citipes* TMP 1982.16.6. The juvenile *Gorgosaurus* is ~12.7% of the body mass of an adult *Gorgosaurus* CMN 2120.

6. Predator-prey mass regressions

To assess the likelihood of *Gorgosaurus* preying on *Citipes*, the influence of body mass on predator-prey mass relationships was investigated in extant terrestrial mammalian and reptilian predators. Maximum and minimum prey mass was compiled for a range of terrestrial reptilian and mammalian predators based on the literature. Data for terrestrial mammals were gathered from Tucker and Rogers (*63*) and those of non-varanid lizards were from Costa et al. (*64*) (see Data S1). Because the latter reported prey size in terms of volume, we transformed body volume into body mass by assuming body density close to that of water (1 ml = 1 g). Information regarding maximum and minimum prey mass and predator mass for extant varanid and crocodylian species was gathered from various sources (*25, 44, 65-83*) and are reported in Data S1. Although Drumheller and Wilberg (*69*) listed the small prey hunted by each crocodilian species, they did not report minimum prey mass; for this reason, we used mass values for equivalent prey species reported in the aforementioned varanid diet literature. It is worth mentioning that the smallest prey reported for large reptiles, particularly crocodylians and large varanids, are not usually ingested by mature predators but by juvenile individuals as these species undergo an ontogenetic dietary shift, feeding on small prey when young and shifting to large prey as they grow (*25, 26, 44-46*).

In order to consider the phylogenetic relationships of predators, phylogenetically-corrected least-squares (PGLS) regressions of maximum and minimum prey mass against predator mass were conducted for reptilian and mammalian predators. Phylogenetic trees of mammals and reptiles were constructed based on Nyakatura and Bininda-Emonds (*84*), Pyron et al. (*85*), and Drumheller and Wilberg (*69*) (figs S4 and S5, Data S2 and S3). Branch length was calculated from divergence time, which was taken from Nyakatura and Bininda-Emonds (*84*), Drumheller and Wilberg (*69*), and TimeTree (timetree.org: data retrieved from August 6th to 13th, 2020), following the procedure of Motani and Schmitz (*86*). Constructed trees are ultrametric. Phylogenetic models with maximum-likelihood estimations of lambda were analyzed with R v.4.04 using the package *caper* v.1.0.1 (*87*). For comparison with PGLS regression, ordinary non-phylogenetic least-squares regressions were performed using IBM SPSS v. 25. As the maximum-likelihood estimations were highest when the lambda values were zero for mammal regressions, this indicates that phylogeny does not affect the regressions (Table S3). In contrast, the maximum-likelihood estimations were highest when the lambda values were greater than zero in reptile regressions, indicating that phylogeny does affect regressions; both PGLS and OLS are reported in Table S3.

The bivariate plot (Figure 5 in main text) demonstrates that ceratopsids (e.g., *Styracosaurus albertensis*, 4370 kg, (*107*)) and hadrosaurids (e.g., *Corythosaurus casuarius*, 3620 kg, (*107*)), the documented diet of adult *Gorgosaurus* (*17*), are consistent with maximum prey size for mammalian predators with a body mass of an adult *Gorgosaurus* but surpass the predicted maximum prey size for similar-sized reptilian predators. However, actual maximum prey size for some extant, large crocodylians plot along the mammalian regression and near ceratopsids and hadrosaurids, revealing that large reptilian predators are capable of capturing very large prey relative to their body size. In comparison, both yearling and adult *Citipes* fall in the lower prey

size range for mammalian predators with a body mass of an adult *Gorgosaurus* and in the upper prey size range for reptilian predators of the size of an adult *Gorgosaurus*. In the context of a juvenile *Gorgosaurus* individual, the bivariate plot shows that juvenile and adult *Citipes* plot in the mid-size prey range for mammalian predators with a body mass of a juvenile *Gorgosaurus* and in the upper range for similar-sized reptilian predators. Dinosaurian megaherbivores plot far above the maximum prey size for either mammalian or reptilian predators of this body mass. These results suggest that *Citipes* could have been a common prey choice for juvenile *Gorgosaurus* individuals but more rarely selected by adult individuals due to its small size, whereas dinosaurian megaherbivores (i.e., hadrosaurids and ceratopsids) could have been preyed upon by adult *Gorgosaurus* individuals but not juvenile individuals.

7. Measurements of specimens

A list of select measurements for the juvenile *Gorgosaurus* and *Citipes* individuals (TMP 2009.12.14) is provided in Table S4.

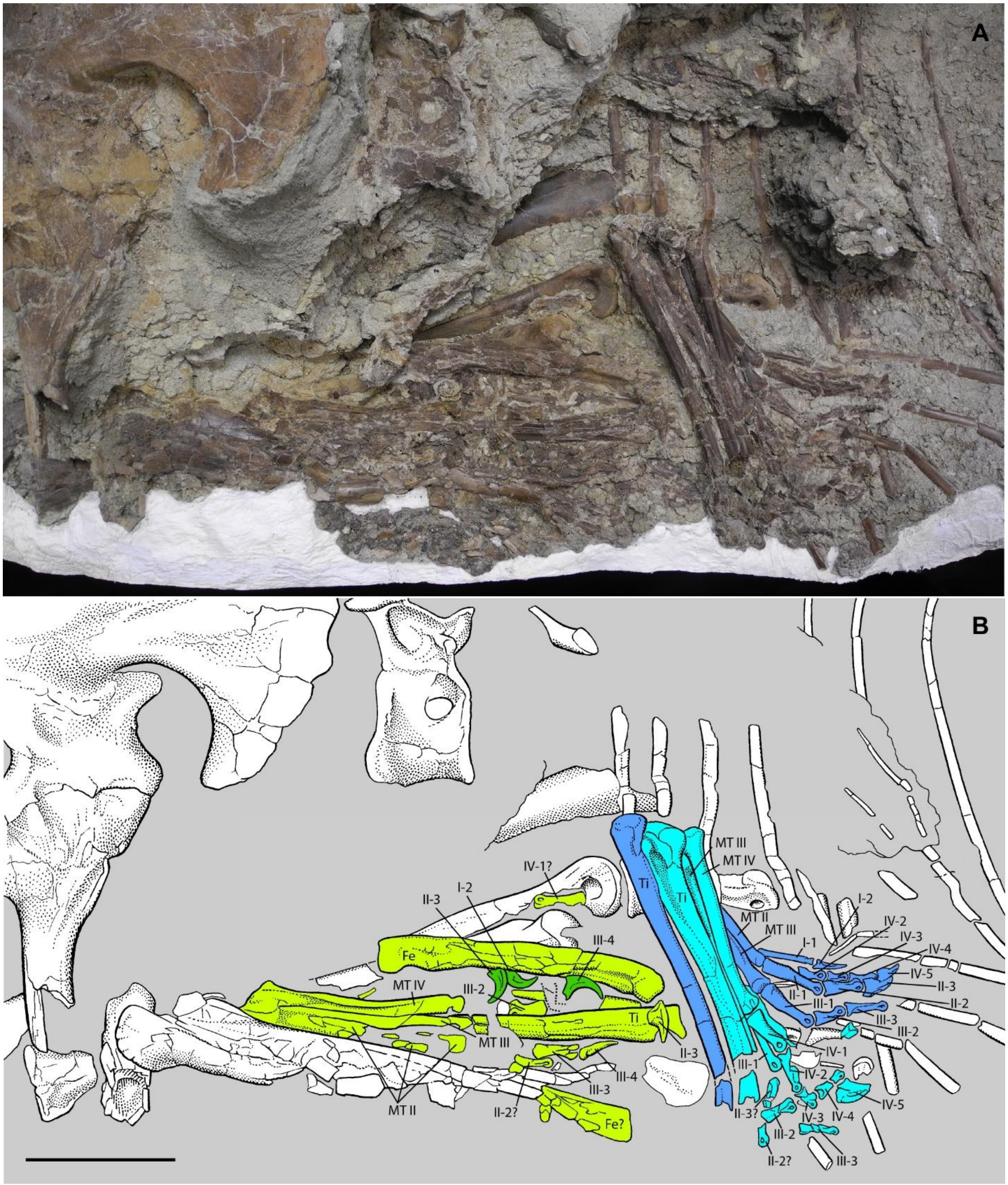

Fig. S1.

Detailed interpretive illustration of the stomach contents of TMP 2009.12.14. Light blue and dark blue colors represent left and right hindlimb elements, respectively, of the anterior *Citipes* individual, the light green color represents hindlimbs of the posterior *Citipes* individual, dark green color represents manual unguals of the posterior *Citipes* individual, and white bones represent elements of the juvenile *Gorgosaurus*. Caudal vertebrae of posterior *Citipes* individual were removed to expose underlying elements. Abbreviations: Fe, femur; Ti, tibia; MT, metatarsal; combination of Roman and Arabic numerals represents phalangeal designation. Scale bar is 10 cm.

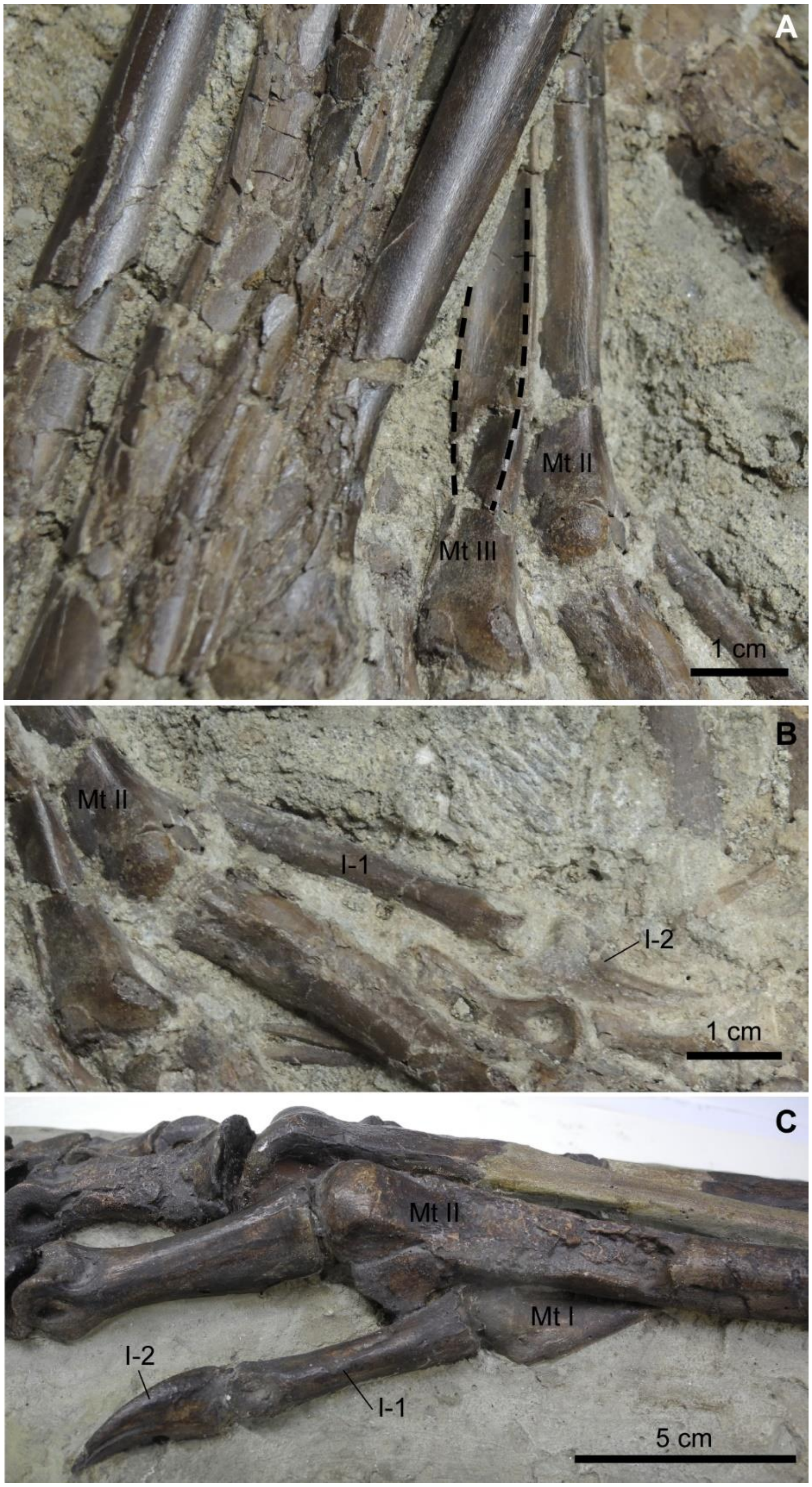

Fig. S2.

Diagnostic taxonomic features present in stomach contents. (A) Anterodistal view of right metatarsus of anterior individual showing the presence of a longitudinal sulcus (delineated by dash lines) on the anterior surface of metatarsal III, diagnostic of *Citipes elegans*. Long, tubular metatarsal I and elongate ungual I-1 of (B) anterior individual and of (C) the caenagnathid *Chirostenotes pergracilis* (cast of CMN 8538).

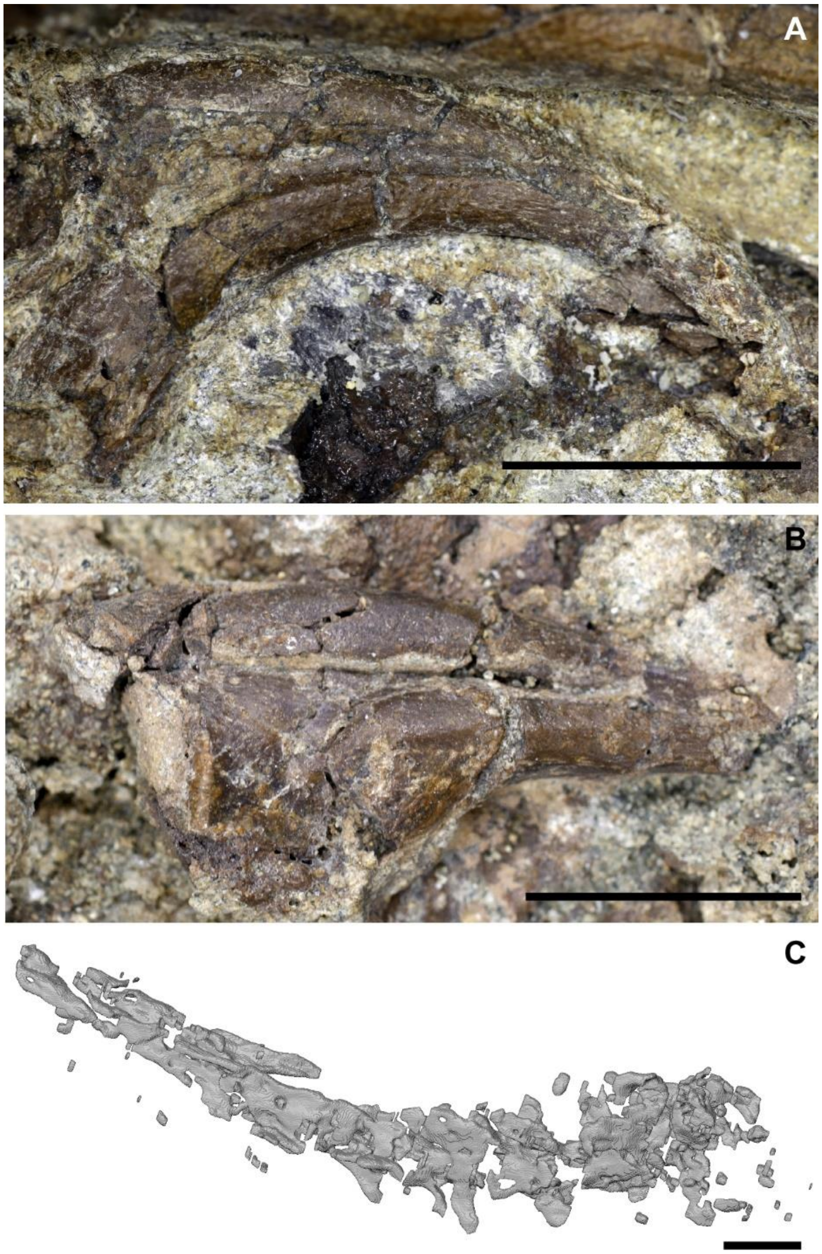

Fig. S3.

Details of select postcranial elements of the posterior *Citipes* individual. (A) Manual ungual III-4 with deep flexor tubercle. (B) Short, robust, gently curved, and proximally swollen pedal ungual II-3. (C) Digital rendering of articulated caudal vertebral series based on CT scans and produced with the software Amira v.2019.1. Scale bars are 1 cm.

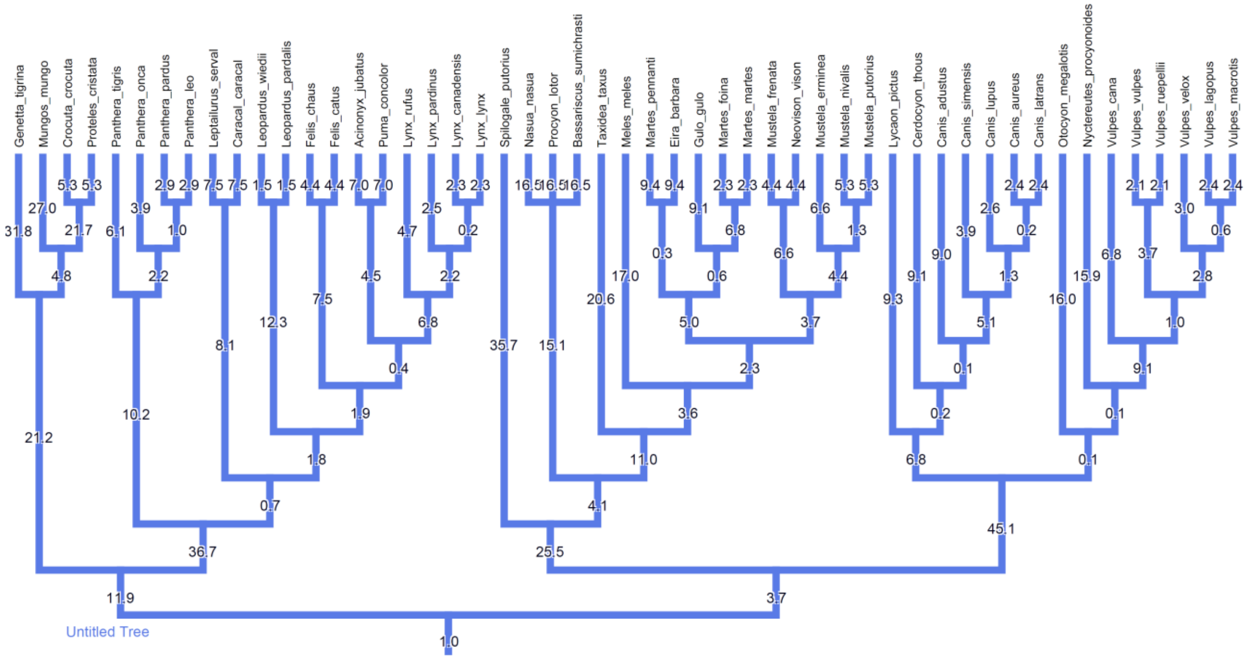

Fig. S4.

Cladogram of extant mammalian predators. Branch length is indicated in black. See Data S2.

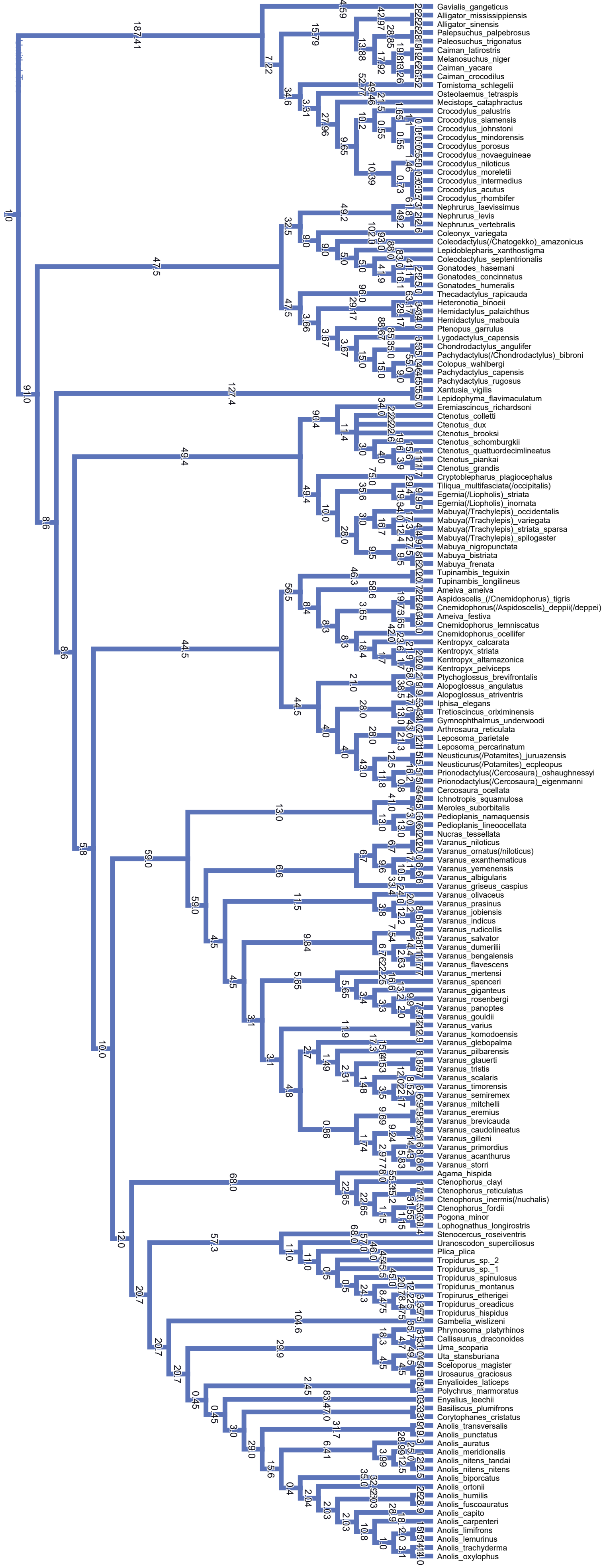

Figure S5.
Cladogram of extant reptilian predators. Branch length is indicated in black. See Data S3.

**Criterion**	**Condition in TMP 2009.12.14**
**Co-allochthonous**: food items and predator co-occur in non-habitat host rock	*Gorgosaurus* and *Citipes* individuals are preserved in fluvial sandstone
**Anatomical position A**: food items enclosed within three-dimensional body cavity of predator	Yes
**Anatomical position B**: food items in appropriate position for stomach	Yes
**Exceptional preservation**: soft tissues (e.g., skin) well-preserved	No
**Size uniformity of food items**	Yes
**Mastication of material**: cleanly sheared leaf margins	Not applicable
**Presence of gastroliths**	No
**Mass mineralogy/sedimentology**: food item mass distinct from surrounding matrix	Absence of mudclasts and coarse particles associated with *Citipes* remains when they are present everywhere else
**Mass margin**: organic envelope or defined margin surrounding mass	No
**Mass shape**: three-dimensional spheroid or oblong mass	Two-dimensional oblong concentration
**Content restricted**: food items localized internally and absent in external matrix	Yes
**Unusual concentration, rarity of food items**	Yes
**Distinct palynomorphs**: palynomorphs present in mass and host rock are distinct	Not applicable
**Acid etching on bone**	Yes

**Geochemical signature**: evidence of stomach enzymes, etc.	Acid etching of bone surface (see text above)
**Dietary items appropriate**	Yes

Table S1.
Criteria supporting identification of stomach contents in TMP 2009.12.14.

**Taxon**	**Femur length (mm)**	**Estimated body mass (kg)**
Gorgosaurus TMP 2009.14.12	543.3	334.4
Adult *Gorgosaurus* (CMN 2120)	1030	2626
Subadult *Gorgosaurus* (TMP 1991.36.500)	650	595.5
Citipes (posterior)	191	11.5
Citipes (anterior)	178*	9.2
Adult *Citipes* (TMP 1982.16.6)	225.9*	19.8

Table S2.

Body mass estimates for *Gorgosaurus* and *Citipes*. Asterisks "*" indicate that femur lengths of anterior and adult *Citipes* are estimated based on length ratios of metatarsal IV/femur of posterior *Citipes* individual (see Table S4).

Group	Model	n	λ	*a*	*b*	R^2
M	OLS: log min prey mass = *a* log predator mass + *b*	48	-	1.142 (p << 0.01)	-3.179 (p <0.01)	0.244
M	OLS: log max prey mass = *a* log predator mass + *b*	51	-	1.165 (p << 0.01)	-0.233 (p = 0.453)	0.250
M	PGLS: log min prey mass = *a* log predator mass + *b*	48	0	1.142 (p << 0.01)	-3.179 (p << 0.01)	0.244
M	PGLS: log max prey mass = *a* log predator mass + *b*	51	0	1.165 (p << 0.01)	-0.233 (p = 0.453)	0.250
R	OLS: log min prey mass = *a* log predator mass + *b*	179	-	0.709 (p << 0.01)	-4.096 (p << 0.01)	0.675
R	OLS: log max prey mass = *a* log predator mass + *b*	179	-	1.043 (p << 0.01)	-1.085 (p << 0.01)	0.877
R	PGLS: log min prey mass = *a* log predator mass + *b*	179	0.593	0.681 (p << 0.01)	-4.244 (p << 0.01)	0.311
R	PGLS: log max prey mass = *a* log predator mass + *b*	179	0.206	0.941 (p << 0.01)	-1.034 (p << 0.01)	0.663

Table S3.

Summary of regression analyses for extant mammalian and reptilian predators. Abbreviations: M, mammalian predators; R, reptilian predators; OLS, ordinary least-squares regression (not phylogenetically-corrected); PGLS, phylogenetically-corrected generalized least-squares regression; λ, phylogenetic signal fitted by maximum likelihood (see text above).

	Gorgosaurus	Anterior *Citipes* - right leg	Anterior *Citipes* - left leg	Posterior *Citipes*	Adult *Citipes* (TMP 1982.16.6)
Skull length (premax-occipital condyle)	493				
Scapulocoracoid length	320+				
Humerus length	138+				
Radius length	75+				
Ulna length	75+				
Metacarpal I	35.5				
Manual Phalanx I-1	61.5*				
Ilium length	485				
Ischium length	330				
Femur length	543.3			191	
Tibia length	614.5	205.2+	176.3+	272	
Fibula length	575				
Metatarsal I	65	21			
Metatarsal II	388	~124.2		116+	152.4***
Metatarsal III	423	~136		145.9	172.2***
Metatarsal IV	398		126.6	135.7	160.5***
Metatarsal V	135				44.3***
I-1	66.2	36.6			
I-2	53.3	18.7			
II-1	102.4	44.4			
II-2	65.2	27.6	20.8+	10.6+	
II-3	61.5	17.2	15+	22.2	
III-1	104	43.5	27.5+		
III-2	70	20	23	24.2	
III-3	51.5	25.5	25.1	30	
III-4	65			25.7	
IV-1	73.3		33	33.7	
IV-2	51.5	23.2	23.9	22.5	
IV-3	33	15	15		
IV-4	21.8	~16.9	18.6		
IV-5	61	15.5	16.6		
Manual I-2				19+	
Manual II-3				22.7	
Manual III-4				19.8-25.4**	

Table S4.

Measurements (in mm) of skeletal elements of *Gorgosaurus* and *Citipes* specimens in TMP 2009.12.14.
+ minimum value
* estimate
** range of estimates because the distal end of the claw is poorly preserved
*** measurements from Funston et al. (*98*)

Data S1. (separate file)

Body masses for extant reptilian and mammalian predators and their respective largest and smallest prey.

Data S2. (separate file)

Mesquite file for regressions of extant mammalian predator data.

Data S3. (separate file)

Mesquite file for regressions of extant reptilian predator data.

REFERENCES AND NOTES

1. S. L. Brusatte, T. D. Carr, The phylogeny and evolutionary history of tyrannosauroid dinosaurs. *Sci. Rep.* **6**, 20252 (2016).

2. S. L. Brusatte, M. A. Norell, T. D. Carr, G. M. Erickson, J. R. Hutchinson, A. M. Balanoff, G. S. Bever, J. N. Choiniere, P. J. Makovicky, X. Xu, Tyrannosaur paleobiology: New research on ancient exemplar organisms. *Science* **329**, 1481–1485 (2010).

3. P. J. Currie, Cranial anatomy of tyrannosaurid dinosaurs from the Late Cretaceous of Alberta, Canada. *Acta Palaeontol. Pol.* **48**, 191–226 (2003).

4. G. M. Erickson, P. J. Makovicky, P. J. Currie, M. A. Norell, S. A. Yerby, C. A. Brochu, Gigantism and comparative life-history parameters of tyrannosaurid dinosaurs. *Nature* **430**, 772–775 (2004).

5. G. F. Funston, M. J. Powers, S. A. Whitebone, S. L. Brusatte, J. B. Scannella, J. R. Horner, P. J. Currie, Baby tyrannosaurid bones and teeth from the Late Cretaceous of western North America. *Can. J. Earth Sci.* **58**, 756–777 (2021).

6. T. D. Carr, Craniofacial ontogeny in Tyrannosauridae (Dinosauria, Coelurosauria). *J. Vert. Pal.* **19**, 497–520 (1999).

7. T. D. Carr, A high-resolution growth series of *Tyrannosaurus rex* obtained from multiple lines of evidence. *PeerJ* **8**, e9192 (2020).

8. G. M. Erickson, S. D. Van Kirk, J. Su, M. E. Levenston, W. E. Caler, D. R. Carter, Bite-force estimation for *Tyrannosaurus rex* from tooth-marked bones. *Nature* **382**, 706–708 (1996).

9. P. M. Gignac, G. M. Erickson, The biomechanics behind extreme osteophagy in *Tyrannosaurus rex. Sci. Rep.* **7**, 2012 (2017).

10. F. Therrien, D. K. Zelenitsky, J. T. Voris, K. Tanaka, Mandibular force profiles and tooth morphology in growth series of Albertosaurus *sarcophagus and Gorgosaurus*

libratus(Tyrannosauridae: Albertosaurinae) provide evidence for an ontogenetic dietary shift in tyrannosaurids. *Can. J. Earth Sci.* **58**, 812–828 (2021).

11. J. T. Voris, D. K. Zelenitsky, F. Therrien, R. C. Ridgely, P. J. Currie, L. M. Witmer, Two exceptionally-preserved juvenile specimens of *Gorgosaurus libratus* (Tyrannosauridae: Albertosaurinae) provide new insight into the timing of ontogenetic changes in tyrannosaurids. *J. Vert. Pal.* **41**, e2041651 (2022).

12. J. O. Farlow, Speculations about the diet and foraging behavior of large carnivorous dinosaurs. *Am. Midl. Nat.* **95**, 186–191 (1976).

13. T. R. Holtz Jr., Theropod guild structure and the tyrannosaurid niche assimilation hypothesis: Implications for predatory dinosaur macroecology and ontogeny in later Late Cretaceous Asiamerica. *Can. J. Earth Sci.* **58**, 778–795 (2021).

14. P. R. Bell, P. J. Currie, Y.-N. Lee, Tyrannosaur feeding traces on *Deinocheirus* (Theropoda:?Ornithomimosauria) remains from the Nemegt Formation (Late Cretaceous), Mongolia. *Cret. Res.* **37**, 186–190 (2012).

15. R. A. DePalma II, D. A. Burnham, L. D. Martin, B. M. Rothschild, P. L. Larson, Physical evidence of predatory behavior in *Tyrannosaurus rex*. *Proc. Natl. Acad. Sci. U.S.A.* **110**, 12560–12564 (2013).

16. D. W. E. Hone, M. Watabe, New information on scavenging and selective feeding behaviour of tyrannosaurids. *Acta Palaeontol. Pol.* **55**, 627–634 (2010).

17. A. R. Jacobsen, Feeding behaviour of carnivorous dinosaurs as determined by tooth marks on dinosaur bones. *Hist. Biol.* **13**, 17–26 (1998).

18. J. E. Martin, A. Hassler, G. Montagnac, F. Therrien, V. Balter, The stability of dinosaur communities before the Cretaceous-Paleogene (K-Pg) boundary: A perspective from southern Alberta using calcium isotopes as a dietary proxy. *GSA Bull.* **134**, 2548–2560 (2022).

19. K. Owocki, B. Kremer, M. Cotte, H. Bocherens, Diet preferences and climate inferred from oxygen and carbon isotopes of tooth enamel of *Tarbosaurus bataar* (Nemegt Formation, Upper Cretaceous, Mongolia). *Palaeogeogr. Palaeocl. Palaeoeco.* **537**, 109190 (2020).

20. D. J. Varricchio, Gut contents from a Cretaceous tyrannosaurid: Implications for theropod dinosaur digestive tracts. *J. Paleo.* **75**, 401–406 (2001).

21. J. E. Peterson, K. N. Daus, Feeding traces attributable to juvenile *Tyrannosaurus rex* offer insight into ontogenetic dietary trends. *PeerJ* **7**, e6573 (2019).

22. R. J. Blumenschine, Carcass consumption sequences and the archaeological distinction of scavenging and hunting. *J. Hum. Evol.* **15**, 639–659 (1986).

23. G. Pérez-Higareda, A. Rangel-Rangel, H. M. Smith, D. Chiszar, Comments on the food and feeding habits of Morelet's crocodile. *Copeia* **1989**, 1039–1041 (1989).

24. S. G. Platt, T. R. Rainwater, S. Snider, A. Garel, T. A. Anderson, S. T. McMurry, Consumption of large mammals by *Crocodylus moreletii*: Field observations of necrophagy and interspecific kleptoparasitism. *Southwest. Nat.* **52**, 310–317 (2007).

25. W. Auffenberg, *The Behavioral Ecology of the Komodo Monitor* (University Press of Florida, 1981).

26. G. Grigg, D. Kirshner, *Biology and Evolution of Crocodylians* (Cornell Univ. Press, 2015).

27. C. G. Farmer, T. J. Uriona, D. B. Olsen, M. Steenblik, K. Sanders, The right-to-left shunt of crocodilians serves digestion. *Physiol. Biochem. Zool.* **81**, 125–137 (2008).

28. L. Xing, P. R. Bell, W. S. Persons IV, S. Ji, T. Miyashita, M. E. Burns, Q. Ji, P. J. Currie, Abdominal contents from two large Early Cretaceous compsognathids (Dinosauria: Theropoda) demonstrate feeding on confuciusornithids and dromaeosaurids. *PLOS ONE* **7**, e44012 (2012).

29. K. Chin, D. A. Eberth, M. H. Schweitzer, T. A. Rando, W. J. Sloboda, J. R. Horner, Remarkable preservation of undigested muscle tissue within a Late Cretaceous tyrannosaurid coprolite from Alberta, Canada. *PALAIOS* **18**, 286–294 (2003).

30. K. Chin, T. T. Tokaryk, G. M. Erickson, L. C. Calk, A king-sized theropod coprolite. *Nature* **393**, 680–682 (1998).

31. J. K. O'Connor, Z. Zhou, The evolution of the modern avian digestive system: Insights from paravian fossils from the Yanliao and Jehol biotas. *Palaeontol.* **63**, 13–27 (2020).

32. W. Ma, M. Pittman, S. Lautenschlager, L. E. Meade, X. Xu, Functional morphology of the oviraptorosaurian and scansoriopterygid skull. *Bull. Am. Mus. Nat. Hist.* **440**, 229–250 (2020).

33. R. F. Ewer, *The Carnivores* (Cornell Univ. Press, 1973).

34. L. D. Mech, *The Wolf: The Ecology and Behavior of An Endangered Species* (University of Minnesota Press, 1970).

35. C. M. Brown, D. C. Evans, N. E. Campione, L. J. O'Brien, D. A. Eberth, Evidence for taphonomic size bias in the Dinosaur Park Formation (Campanian, Alberta), a model Mesozoic terrestrial alluvial-paralic system. *Palaeogeogr. Palaeocli. Palaeoeco* **372**, 108–122 (2013).

36. C. M. Brown, D. C. Evans, M. J. Ryan, A. P. Russell, New data on the diversity and abundance of small-bodied ornithopods (Dinosauria, Ornithischia) from the Belly River Group (Campanian) of Alberta. *J. Vert. Pal.* **33**, 495–520 (2013).

37. D. Codron, C. Carbone, M. Clauss, Ecological interactions in dinosaur communities: Influences of small offspring and complex ontogenetic life histories. *PLOS ONE* **8**, e77110 (2013).

38. J. O. Farlow, T. R. Holtz Jr., The fossil record of predation in dinosaurs. *The Pal. Soc. Papers* **8**, 251–266 (2002).

39. P. J. Currie, Possible evidence of gregarious behavior in tyrannosaurids. *Gaia* **15**, 271–277 (2000).

40. K. Schroeder, S. K. Lyons, F. A. Smith, The influence of juvenile dinosaurs on community structure and diversity. *Science* **371**, 941–944 (2021).

41. J. Sánchez-Hernández, A. D. Nunn, C. E. Adams, P.-A. Amundsen, Causes and consequences of ontogenetic dietary shifts: A global synthesis using fish models. *Biol. Rev.* **94**, 539–554 (2019).

42. T. Schellekens, A. M. de Roos, L. Persson, Ontogenetic diet shifts result in niche partitioning between two consumer species irrespective of competitive abilities. *Am. Nat.* **176**, 625–637 (2010).

43. E. E. Werner, J. F. Gilliam, The ontogenetic niche and species interactions in size-structured populations. *Annu. Rev. Ecol. Syst.* **15**, 393–425 (1984).

44. D. Purwandana, A. Ariefiandy, M. J. Imansyah, A. Seno, C. Ciofi, M. Letnic, T. S. Jessop, Ecological allometries and niche use dynamics across Komodo dragon ontogeny. *Sci. Nat.* **103**, 27 (2016).

45. H. B. Cott, Scientific results of an inquiry into the ecology and economic status of the Nile Crocodile (*Crocodius niloticus*) in Uganda and northern Rhodesia. *J. Zool.* **29**, 211–356 (1961).

46. P. M. Gignac, G. M. Erickson, Ontogenetic changes in dental form and tooth pressures facilitate developmental niche shifts in American alligators. *J. Zool.* **295**, 132–142 (2015).

47. P. Dodson, Functional and ecological significance of relative growth in *Alligator*. *J. Zool.* **175**, 315–355 (1975).

48. G. M. Erickson, P. M. Gignac, A. K. Lappin, K. A. Vliet, J. D. Brueggen, G. J. W. Webb, A comparative analysis of ontogenetic bite-force scaling among Crocodylia. *J. Zool.* **292**, 48–55 (2014).

49. G. M. Erickson, A. K. Lappin, K. A. Vliet, The ontogeny of bite-force performance in American alligator (*Alligator mississippiensis*). *J. Zool.* **260**, 317–327 (2003).

50. P. M. Gignac, G. M. Erickson, Ontogenetic bite-force modeling *of Alligator mississippiensis*: Implications for dietary transitions in a large-bodied vertebrate and the evolution of crocodylian feeding. *J. Zool.* **299**, 229–238 (2016).

51. C. Carbone, G. M. Mace, S. C. Roberts, D. W. Macdonald, Energetic constraints on the diet of terrestrial carnivores. *Nature* **402**, 286–288 (1999).

52. E. J. Rayfield, Cranial mechanics and feeding in *Tyrannosaurus rex*. *Proc. R. Soc. Lond. B Bio.* **271**, 1451–1459 (2004).

53. E. Snively, D. M. Henderson, D. S. Phillips, Fused and vaulted nasals of tyranosaurid dinosaurs: Implications for cranial strength and feeding mechanics. *Acta Palaeontol. Pol.* **51**, 435–454 (2006).

54. F. Therrien, D. M. Henderson, C. B. Ruff, "Bite me: Biomechanical models of theropod mandibles and implications for feeding behavior," in *The Carnivorous Dinosaurs*, K. Carpenter, Ed. (Indiana Univ. Press, 2005), pp. 179–237.

55. K. Tanaka, D. K. Zelenitsky, J. Lü, C. L. DeBuhr, L. Yi, S. Jia, F. Ding, M. Xia, D. Liu, C. Shen, R. Chen, Incubation behaviours of oviraptorosaur dinosaurs in relation to body size. *Biol. Lett.* **14**, 20180135 (2018).

56. H. N. Woodward, K. Tremaine, S. A. Williams, L. E. Zanno, J. R. Horner, N. Myhrvold, Growing up *Tyrannosaurus rex*: Osteohistology refutes the pygmy "*Nanotyrannus*" and supports ontogenetic niche partitioning in juvenile *Tyrannosaurus*. *Sci. Adv.* **6**, eaax6250 (2020).

57. A. J. Rowe, E. Snively, Biomechanics of juvenile tyrannosaurid mandibles and their implications for bite force: Evolutionary biology. *Anat. Rec.* **305**, 373–765 (2022).

58. E. Johnson-Ransom, F. Li, X. Xu, R. Ramos, A.J. Midzuk, U. Thon, K. Atkins-Weltman, E. Snively, Comparative cranial biomechanics reveal that Late Cretaceous tyrannosaurids exerted relatively greater bite force than in early-diverging tyrannosauroids. *Anat. Rec.* (2023).

59. S. L. Brusatte, A. Averianov, H.-D. Sues, A. Muir, I. B. Butler, New tyrannosaur from the mid-Cretaceous of Uzbekistan clarifies evolution of giant body sizes and advanced senses in tyrant dinosaurs. *Proc. Natl. Acad. Sci. U.S.A.* **113**, 3447–3452 (2016).

60. S. J. Nesbitt, R. K. Denton Jr., M. A. Loewen, S. L. Brusatte, N. D. Smith, A. H. Turner, J. I. Kirkland, A. T. McDonald, D. G. Wolfe, A mid-Cretaceous tyrannosauroid and the origin of North American end-Cretaceous dinosaur assemblages. *Nat. Ecol. Evol.* **3**, 892–899 (2019).

61. L. E. Zanno, R. T. Tucker, A. Canoville, H. M. Avrahami, T. A. Gates, P. J. Makovicky, Diminutive fleet-footed tyrannosauroid narrows the 70-million-year gap in the North American fossil record. *Comm. Biol.* **2**, 64 (2019).

62. M. D. D'Emic, P. M. O'Connor, R. S. Sombathy, I. Cerda, T. R. Pascucci, D. J. Varricchio, D. Pol, A. Dave, R. A. Coria, K. A. Curry Rogers, Developmental strategies underlying gigantism and miniaturization in non-avialan theropod dinosaurs. *Science* **379**, 811–814 (2023).

63. M. A. Tucker, T. L. Rogers, Examining the prey mass of terrestrial and aquatic carnivorous mammals: Minimum, maximum and range. *PLOS ONE* **9**, e106402 (2014).

64. G. C. Costa, L. J. Vitt, E. R. Pianka, D. O. Mesquita, G. R. Colli, Optimal foraging constrains macroecological patterns: Body size and dietary niche breadth in lizards. *Glob. Ecol. Biogeogr.* **17**, 670–677 (2008).

65. F. M. Angelici, L. Luiselli, Aspects of the ecology of *Varanus niloticus* (Reptilia, Varanidae) in southeastern Nigeria, and their contribution to the knowledge of the evolutionary history of *V. niloticus* species complex. *Revue d'Ecologie* **54**, 29–42 (1999).

66. W. Auffenberg, in *Gray's Monitor Lizard* (University Press of Florida, 1988), p. 1101.

67. S. L. Cross, M. D. Craig, S. Tomlinson, P. W. Bateman, I don't like crickets, I love them: Invertebrates are an important prey source for varanid lizards. *J. Zool.* **310**, 323–333 (2020).

68. K. Dalhuijsen, W. R. Branch, G. J. Alexander, A comparative analysis of the diets of Varanus *albigularis* and *Varanus* niloticusin South Africa. *Afr. Zool.* **49**, 83–93 (2014).

69. S. K. Drumheller, E. W. Wilberg, A synthetic approach for assessing the interplay of form and function in the crocodyliform snout. *Zool. J. Linn. Soc.* **188**, 507–521 (2020).

70. B. E. Emmanuel, The fishery and bionomics of the swimming crab, *Callinectes amnicola* (DeRocheburne, 1883) from a tropical lagoon and its adjacent creek, southwest Nigeria. *J. Fish. Aqu. Sci.* **3**, 114–125 (2008).

71. C. D. James, J. B. Losos, D. R. King, Reproductive biology and diets of goannas (Reptilia: Varanidae) from Australia. *J. Herpetol.* **26**, 128–136 (1992).

72. D. King, B. Green, Notes on diet and reproduction of the Sand Goanna, Varanus gouldii rosenbergi. *Copeia* **1979**, 64–70 (1979).

73. Encyclopedia of Life online database, https://eol.org/.

74. J. B. Losos, H. W. Greene, Ecological and evolutionary implications of diet in monitor lizards. *Biol. J. Linn. Soc.* **35**, 379–407 (1988).

75. Z. N. Mahmoud, D. A. A. Elnaeem, Hematocrit and blood volume in the common African toad (*Bufo regularis*). *Herpet. J.* **1**, 51–52 (1986).

76. P. J. Mayes, G. G. Thompson, P. C. Withers, Diet and foraging behaviour of the semi-aquatic *Varanus mertensi* (Reptilia:Varanidae). *Wildlife Res.* **32**, 67–74 (2005).

77. E. J. O'Gorman, D. W. E. Hone, Body size distribution of the dinosaurs. *PLOS ONE* **7**, e51925 (2012).

78. J. H. Pascoe, R. C. Mulley, R. Spencer, R. Chapple, Diet analysis of mammals, raptors and reptiles in a complex predator assemblage in the Blue Mountains, eastern Australia. *Aust. J. Zool.* **59**, 295–301 (2011).

79. E. R. Pianka, D. King, *Varanoid Lizards of the World* (Indiana Univ. Press, 2004).

80. K. M. M. Rahman, I. I. Rakhimov, Activity patterns and feeding ecology of the semi-aquatic *Varanus flavescens* (Reptilia: Varanidae). *Russ. J. Herp.* **26**, 91–97 (2019).

81. R. Shine, Food habits, habitats and reproductive biology of four sympatric species of varanid lizards in tropical Australia. *Herpetologica* **42**, 346–360 (1986).

82. D. R. Sutherland, Dietary niche overlap and size partitioning in sympatric varanid lizards. *Herpetologica* **67**, 146–153 (2011).

83. L. Trutnau, R. Sommerlad, *Crocodilians: Their Natural History & Captive Husbandry* (Edition Chimaira, 2006).

84. K. Nyakatura, O. R. P. Bininda-Emonds, Updating the evolutionary history of Carnivora (Mammalia): A new species-level supertree complete with divergence time estimates. *BMC Biol.* **10**, 12 (2012).

85. R. A. Pyron, F. T. Burbrink, J. J. Wiens, A phylogeny and revised classification of Squamata, including 4161 species of lizards and snakes. *BMC Evol. Biol.* **13**, 93 (2013).

86. R. Motani, L. Schmitz, Phylogenetic versus functional signals in the evolution of form-function relationships in terrestrial vision. *Evolution* **65**, 2245–2257 (2011).

87. D. Orme, R. Freckleton, G. Thomas, T. Petzoldt, S. Fritz, N. Isaac, W. Pearse, CAPER: Comparative analyses of phylogenetics and evolution in R. R package version 1.0.1 (2018); https://cran.rproject.org/web/packages/caper/index.html.

88. D. A. Eberth, "The geology," in *Dinosaur Provincial Park: A Spectacular Ancient Ecosystem Revealed*, P. J. Currie, E. B. Koppelhus, Eds. (Indiana Univ. Press, 2005), pp. 54–82.

89. J. Ramezani, T. L. Beveridge, R. R. Rogers, D. A. Eberth, E. M. Roberts, Calibrating the zenith of dinosaur diversity in the Campanian of the Western Interior Basin by CA-ID-TIMS U–Pb geochronology. *Sci. Rep.* **12**, 16026 (2022).

90. T. M. Cullen, L. E. Zanno, D. W. Larson, E. Todd, P. J. Currie, D. C. Evans, Anatomical, morphometric, and stratigraphic analyses of theropod biodiversity in the Upper Cretaceous (Campanian) Dinosaur Park Formation. *Can. J. Earth Sci.* **58**, 870–884 (2021).

91. P. J. Currie, D. A. Russell, Osteology and relationships of Chirostenotes pergracilis (Saurischia, Theropoda) from the Judith River (Oldman) Formation of Alberta, Canada. *Can. J. Earth Sci.* **25**, 972–986 (1988).

92. C. M. Sternberg, Two new theropod dinosaurs from the Belly River Formation of Alberta. *Can. Field Nat.* **46**, 99–105 (1932).

93. C. M. Brown, D. R. Greenwood, J. E. Kalyniuk, D. R. Braman, D. M. Henderson, C. L. Greenwood, J. F. Basinger, Dietary palaeoecology of an Early Cretaceous armoured dinosaur (Ornithischia; Nodosauridae) based on floral analysis of stomach contents. *Roy. Soc. Open Sci.* **7**, 200305 (2020).

94. T. D. Carr, D. J. Varricchio, J. C. Sedlmayr, E. M. Roberts, J. R. Moore, A new tyrannosaur with evidence for anagenesis and crocodile-like facial sensory system. *Sci. Rep.* **7**, 44942 (2017).

95. J. T. Voris, D. K. Zelenitsky, F. Therrien, P. J. Currie, Reassessment of a juvenile *Daspletosaurus* from the Late Cretaceous of Alberta, Canada with implications for the identification of immature tyrannosaurids. *Sci. Rep.* **9**, 17801 (2019).

96. D. A. Russell, Tyrannosaurs from the Late Cretaceous of western Canada. *Natl. Mus. Nat. Sci. Publ. Paleontol.* **1**, 1–34 (1970).

97. A. Chinsamy-Turan, *The Microstructure of Dinosaur Bones: Deciphering Biology Through Fine Scale Techniques* (John Wiley & Sons, 2005).

98. G. F. Funston, P. J. Currie, M. E. Burns, New elmisaurine specimens from North America and their relationship to the Mongolian *Elmisaurus rarus*. *Acta Palaeontol. Pol.* **61**, 159–173 (2016).

99. R. C. Ridgely, L. M. Witmer, Dead on arrival: Optimizing CT data acquisition of fossils using modern hospital CT scanners. *J. Vert. Pal. Abst. with Prog.* **26**, 115A (2006).

100. W. S. Persons IV, P. J. Currie, M. A. Norell, Oviraptorosaur tail forms and functions. *Acta Palaeontol. Pol.* **59**, 553–567 (2014).

101. C. O. D. C. Diefenbach, Gastric function in *Caiman crocodilus* (Crocodylia: Reptilia) - II. Effects of temperature on pH and proteolysis. *Comp. Biochem. Phys. A* **51**, 267–274 (1975).

102. J. F. Anderson, A. Hall-Martin, D. A. Russell, Long-bone circumference and weight in mammals, birds and dinosaurs. *J. Zool.* **207**, 53–61 (1985).

103. N. E. Campione, D. C. Evans, C. M. Brown, M. T. Carrano, L. Revell, Body mass estimation in non-avian bipeds using a theoretical conversion to quadruped stylopodial proportions. *Methods Ecol. Evol.* **5**, 913–923 (2014).

104. P. Christiansen, R. A. Fariña, Mass prediction in theropod dinosaurs. *Hist. Biol.* **16**, 85–92 (2004).

105. N. E. Campione, D. C. Evans, The accuracy and precision of body mass estimation in non-avian dinosaurs. *Biol. Rev.* **95**, 1759–1797 (2020).

106. E. Snively, H. O'Brien, D. M. Henderson, H. Mallison, L. A. Surring, M. E. Burns, T. R. Holtz Jr., A. P. Russell, L. M. Witmer, P. J. Currie, S. A. Hartman, J. R. Cotton, Lower rotational inertia and larger leg muscles indicate more rapid turns in tyrannosaurids than in other large theropods. *PeerJ* **7**, e6432 (2019).

107. N. E. Campione, D. C. Evans, A universal scaling relationship between body mass and proximal limb bone dimensions in quadrupedal terrestrial tetrapods. *BMC Biol.* **10**, 60 (2012).
